# Lesser Tuberosity Avulsion Fracture in an 11-Year-Old Baseball Player due to Batting

**DOI:** 10.1155/2021/2396200

**Published:** 2021-11-16

**Authors:** Ryan Cheng, Jay Moran, Samantha Smith, Don Li, Christopher A. Schneble, Shin Mei Chan, Elizabeth C. Gardner

**Affiliations:** ^1^Department of Orthopaedics and Rehabilitation, Yale University School of Medicine, New Haven, CT, USA; ^2^Orthopedic Surgery, Hospital for Special Surgery-Weil-Cornell Medical School, New York, NY, USA

## Abstract

**Case:**

We report an 11-year-old male who sustained a lesser tuberosity avulsion fracture of the proximal humerus upon making contact with a baseball during an at-bat. This injury was neglected for 14 months and was eventually detected by an axillary radiograph and subsequent MRI. He successfully underwent an open surgical repair and regained full range of motion and level of activity at 1-year follow-up.

**Conclusion:**

In pediatric baseball players, lesser tuberosity avulsion fractures may occur upon striking a baseball with a bat. Even after being neglected for several months, these injuries can be treated successfully with an open surgical repair.

## 1. Introduction

Lesser tuberosity avulsion fractures in children and adolescents are relatively rare, with only a few cases reported in the literature [1–3]. Though most of these injuries tend to occur in 12 to 14-year-olds, incidents have been reported in patients as young as 8 years of age, with a predilection towards males [4–10]. These sports-related injuries have been reported in hockey, lacrosse, baseball, football, wrestling, and soccer and commonly occur from a contact mechanism of injury [10–12]. In Little League baseball, lesser tuberosity avulsion fractures have been reported in pitchers and are associated with poor pitching mechanics which place repetitive stress on the shoulder [10, 13].

In the pediatric population, lesser tuberosity avulsion fractures are often missed and can have unspecific findings on both physical examination and radiographic imaging [1–3, 8, 10, 14–16]. Despite these injuries being readily identified on magnetic resonance imaging (MRI), the varying clinical presentations associated with this injury can make it difficult to determine whether MRI or further workup is required [1, 2]. As a result, these injuries are commonly misdiagnosed as strains and are discovered weeks to months after the initial incident [2, 8, 17–20]. Understanding the various mechanisms of injury allows for a better understanding of patient presentations, prevents prolonged shoulder impairment, and enables a quicker return to activity.

This particular report describes the case of an 11-year-old patient who sustained a lesser tuberosity avulsion fracture from hitting a baseball during a Little League Baseball game.

## 2. Case Report

**Figure 1:**
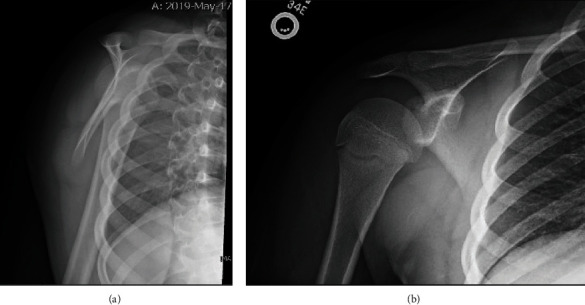
(a) Scapular X-ray taken during patient's first visit to emergency department. (b) Anteroposterior X-ray taken during patient's first visit to emergency department.

An 11-year-old male presented to the emergency department after injuring his shoulder in a baseball game. The patient expressed that he felt a “pop” in his right shoulder while swinging a baseball bat in a Little League game. He denied both a direct blow and any other trauma. On physical exam, he had full, pain-free passive range of motion of the right shoulder but experienced pain with active range of motion, particularly with internal rotation against resistance. Scapular Y and standard anteroposterior shoulder radiographs were obtained and read as negative (Figures 1(a) and 1(b)). He was later discharged with a muscle strain.

Over the course of the following year, the patient had continued shoulder pain and tightness and was thus unable to throw the ball effectively. As a result, he switched positions from the 3^rd^ base to the 1^st^ base to limit the amount of stress placed on his injured shoulder. Despite this transition, he repeatedly experienced episodes of his right shoulder “popping,” which were accompanied by pain.

**Figure 2:**
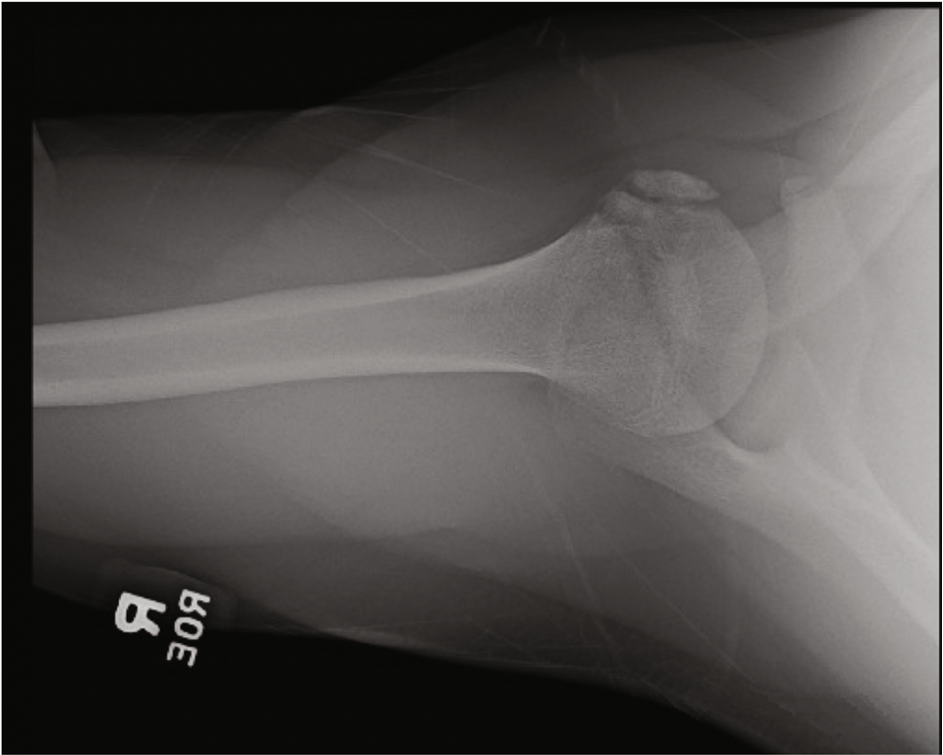
Axillary X-ray reveals age indeterminate fracture from the humeral head at the time of outpatient follow-up one year after injury.

**Figure 3:**
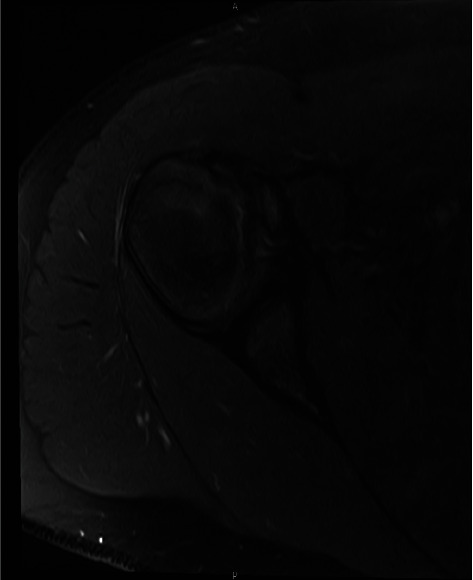
Axial proton density fat saturation image demonstrating the nonunited lesser tuberosity fragment without surrounding edema.

**Figure 4:**
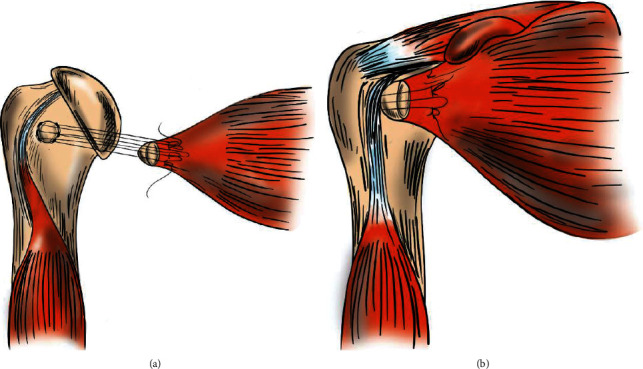
(a) Anteroposterior view of suture placement for avulsion fracture repair; (b) finished repair.

Upon presenting to our institution 14 months postinjury, the patient was otherwise healthy and axillary X-rays demonstrated an age indeterminate fracture of the humeral head (Figure 2). Subsequent MRI showed heterogeneous signal and thickening of subscapularis fibers at the lesser tuberosity attachment as well as osseous fragmentation (Figure 3). These findings were suggestive of a prior avulsion fracture of the lesser tuberosity with interstitial tearing of the inferior subscapularis fibers. Surgery was recommended, and the patient and his family agreed to proceed accordingly. Repair of the subscapularis tendon was performed through the deltopectoral approach (Figures 4(a) and 4(b)). Four Mason-Allen sutures were used to anchor and stabilize the subscapularis tendon with excellent compression. The fracture fragment was tied down to the lateral aspect of the lesser tuberosity and attached to the periosteum with nonabsorbable Ethibond sutures. After the operation, the patient did not stay in the hospital. He was immobilized in a sling for 6 weeks and then rehabbed using a subscapularis tendon repair protocol. The patient had full range of motion at the 3-month follow-up appointment. At the 1-year follow-up, he was asymptomatic, had full range of motion, noted no feelings of locking, and was able to return to throwing and all baseball activities.

## 3. Discussion

Lesser tuberosity avulsion fractures of the proximal humerus are rare injuries in children and adolescents [1–3]. These misdiagnosed injuries typically present as unspecific acute or chronic anterior shoulder pain, making them difficult to recognize on physical exam [3]. The mechanisms that lead to lesser tuberosity avulsion fractures can act a secondary sign for injury recognition and are important for clinical awareness. In this report, we describe a neglected lesser tuberosity fracture that occurred from a bat swinging mechanism in a Little League Baseball pediatric patient. To our knowledge, this mechanism of injury has not been reported in the literature.

Lesser tuberosity avulsion injuries have been previously identified in Little League pitchers [10]. Risk factors include poor pitching mechanics, increased number of curveballs thrown, and increased pitch counts as these variables may intensify the stress that throwing places on the shoulder and leave the lesser tuberosity at greater risk of avulsion [10, 21]. However, the presented case highlights an injury from a batting mechanism in a Little League baseball patient. We hypothesize that the mechanism of this injury may have resulted from external rotation of the humerus in an abducted position and eccentric contraction of the subscapularis upon striking a baseball with a bat. Similar to those who sustain avulsion injuries during contact sports, patients who are injured while batting could present with a sudden, rather than gradual, onset of pain [10]. We recommend further diagnostic workup if a pediatric patient describes a similar course of injury. In addition to Little League pitching, our reported bat swinging mechanism may be the first in developing more focused injury prevention efforts.

Lesser tuberosity avulsion fractures typically go, on average, 2 months (interquartile ranges: 1 month–7 months) before being diagnosed [22]. In our patient, the avulsion of the lesser tuberosity was diagnosed 14 months after the initial injury. Prompt diagnosis of lesser tuberosity avulsion fractures is advantageous, as very few patients are able to regain full range of motion and strength with nonoperative treatment alone [1, 2, 11, 13, 22, 23]. Moreover, timely diagnosis, through the use of MRI, may decrease shoulder impairment and enable continued participation in sports. While reports have showed that delayed treatment may not confer long term sequalae, some have suggested that delayed diagnoses of these injuries may predispose athletes to concomitant injuries, as there is diminished subscapularis strength and anterior shoulder instability [9, 11, 22, 24].

The detection of these injuries without the use of MRI has proven to be quite difficult because the majority of these injuries are not obvious on scapular Y and anteroposterior view radiographs. However, axillary radiographs have been shown to successfully identify many of these injuries [1, 2, 22, 25, 26]. Early MRI has been shown to have a higher sensitivity (0.95) compared to anteroposterior and axillary radiographs (0.16) in detecting lesser tuberosity avulsion fractures [1, 2, 22, 25, 26]. In our patient, axillary X-rays 14 months after the injury demonstrated the avulsion of the lesser tuberosity (Figure 2), whereas anteroposterior and scapular Y radiographs taken at patient's first emergency department visit failed to reveal the fracture (Figures 1(a) and 1(b)). His subsequent shoulder MRI, taken less than a week after the axillary X-rays identified the injury, ultimately revealed heterogeneous signal in the interior fibers of the subscapularis attachment on the lesser tuberosity with osseous fragmentation (Figure 3). If a lesser tuberosity avulsion fracture is suspected, an MRI should be considered for further workup, as demonstrated in our case.

Many pediatric patients who have elected to proceed with nonoperative care have reported the development of chronic shoulder pain, exostoses at the avulsion site, as well as inhibited internal rotation stemming from subscapularis muscle degeneration [1, 2]. Therefore, surgical intervention should be considered an effective method of treating lesser tuberosity avulsion fractures, including those that are only slightly displaced, as these fragments still pose a risk of anteromedial impingement, malunion, nonunion, and may limit shoulder strength and mobility [8]. Nevertheless, nonoperative treatment can be trialed for injuries with minimally displaced fragments or fragments that still retain adequate internal rotation [10]. When determining the appropriate course of treatment and the degree of compromise to subscapularis function, patient's athletic demands should also be taken into account. If persistent pain continues after 12 to 18 months of nonoperative care, this may serve as an additional sign to consider operative treatment [10]. In our case, surgical treatment with an open repair showed excellent results and return to full athletic function when conservative treatment failed.

In conclusion, lesser tuberosity avulsion fractures are difficult to diagnose due to the unspecific physical exam and imaging findings. The reported injury in this case occurred through a unique bat swinging mechanism in Little League baseball. The lesser tuberosity avulsion injury was eventually noticed on both MRI and axillary X-rays as opposed to anteroposterior and scapular X-rays [1, 2, 22, 25, 26]. Physicians should use this report to recognize this unique mechanism of injury and patient presentation to prevent delayed diagnoses in the future.

## Data Availability

No data were used to support this study.
